# Female-specific anti-ageing effects of TRF2 inhibition in skeletal myofibres

**DOI:** 10.1098/rstb.2025.0336

**Published:** 2026-11-12

**Authors:** Maria Sol Jacome Burbano, Jerome D. Robin, Marjorie Martin, Julien Gondin, Mélanie Pousse, Jean-Luc Thomas, Serge Bauwens, Marco Raffaelli, Emmanuelle Girard, Peggy Del Carmine, Edwige Belotti, Pauline Billard, Tiphaine Dorel, Laurent Schaeffer, Eric Gilson

**Affiliations:** ^1^ Côte d'Azur University, CNRS, Inserm, IRCAN, Faculty of Medicine, Nice, France; ^2^ Department of Genetics, CHU, IHU RespirERA, Nice, France; ^3^ Pôle Sino-Français de Recherches en Sciences du Vivant Et Génomique, RuiJin Hospital, Shanghai Jiao Tong University School of Medicine, CNRS, Inserm, Université Côte d'Azur, Shanghai, People's Republic of China; ^4^ Institut NeuroMyoGène, Unité Physiopathologie et Génétique du Neurone et du Muscle, Université Claude Bernard Lyon 1, CNRS UMR 5261, Inserm U1315, Univ. Lyon, Lyon, France; ^5^ Centre De Biotechnologie Cellulaire, Hospices Civils De Lyon, Lyon, France; ^6^ Aix Marseille Univ, MMG, Marseille Medical Genetics U1251, 13005 Marseille, France; ^7^ Plateau de Biologie Expérimentale de la Souris (PBES), Ecole Normale Supérieure de Lyon, Université Lyon1, CNRS UMS3444 INSERM US8, 69007, Lyon, France

**Keywords:** telomere, ageing, TRF2, skeletal muscle

## Abstract

Aged female mice with skeletal muscle-specific deletion of *Terf2*, which encodes the telomere-protective factor TRF2, display a remarkably young-like muscle phenotype including reduced myofibre atrophy, preserved muscle strength, absence of regenerative nuclear markers and modest lifespan extension. This phenotype occurs without evidence of telomere deprotection but is associated with mild pro-oxidant signalling; hence, we propose that age-associated TRF2 downregulation induces a hormetic response that benefits skeletal muscle function during ageing.

This article is part of the theme issue ‘Developmental circuits in ageing’.

## 1. Introduction

The progressive shortening of telomeric DNA is a developmentally regulated process that can drive senescence in dividing cells [1]. Although telomeres were long considered irrelevant to post-mitotic ageing, accumulating evidence challenges this view: cardiomyocytes show telomere dysfunction and senescence during normal ageing [2], together with reduced levels of the telomere-protective factor TRF2 in pathological conditions [3]. TRF2 expression also declines in skeletal muscle from early adulthood onwards [4]. In both human myotubes and mouse myofibres, we previously showed that TRF2 loss does not cause overt telomere deprotection but instead triggers mitochondrial dysfunction and increased reactive oxygen species (ROS) production [4]. In TRF2-compromised myofibres, telomeres are maintained by an alternative protection mechanism that depends on non-canonical, oxidative-stress-activated functions of FOXO3a [5]. Here, we investigate the long-term consequences of TRF2 inactivation in mature mouse myofibres (human skeletal actin (HSA)*-*Cre^+/−^; *Terf2*^flox/flox^) [4].

## 2. Results and discussion

Muscle regeneration was assessed by analysing myonuclear positioning as an indicator of both ongoing and historical regenerative activity [1,2]. While at young ages, most nuclei in the gastrocnemius muscles of the three mouse strains were peripheral, at old ages, HSA-Cre^+/–^ and *Terf2*^flox/flox^ had 5% central nuclei, indicating regeneration events probably owing to age-related muscle injuries. By contrast, HSA-Cre^+/–^, *Terf2*^flox/flox^ mice did not present increased central nuclei with ageing (figure 1A,B; supplementary material, figure S1).

**Figure 1.**
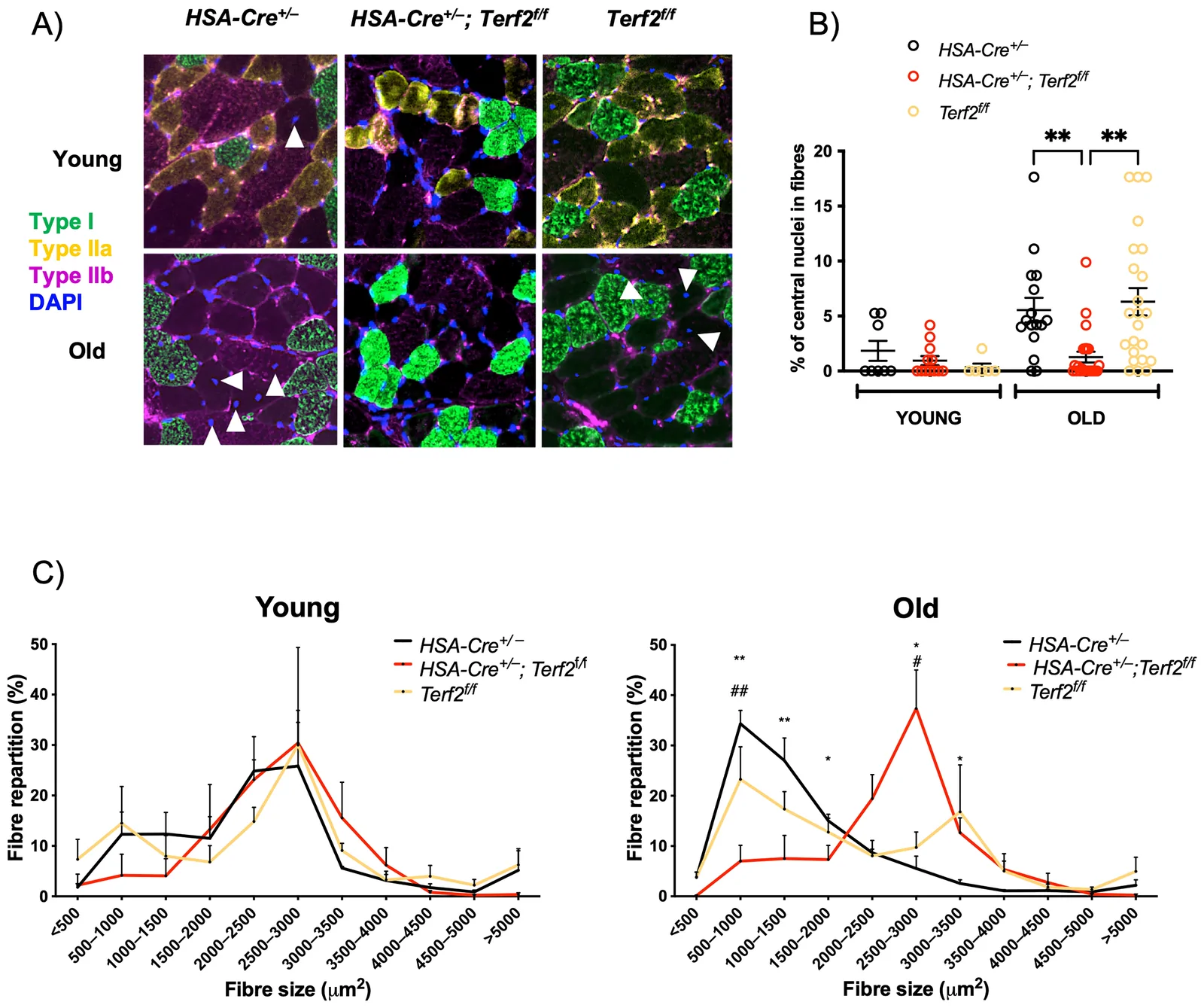
HSA-Cre^+/–^; *Terf2*^f/f^ mice do not undergo the skeletal muscle fibre ageing-related switch. (A) Representative images of gastrocnemius fibre type characterization (type I: green, type IIa: yellow and type IIb: pink) and nuclei 4′,6-diamidino-2-phenylindole (DAPI) staining (in blue). Young (1.5 months) versus old (15 months) mice were compared for three different genotypes: HSA-Cre^+/–^ (young: *n* = 3, old *n* = 10), HSA-Cre^+/–^; *Terf2*^f/f^ (young: *n* = 4, old *n* = 12) and *Terf2*^f/f^ (young: *n* = 2, old *n* = 12). Nucleus position was determined as peripherical or central. (B) Quantification of the percentage of nuclei with central localization in gastrocnemius type II fibres in young and old mice. Statistical analyses were performed using Kruskal–Wallis’ multiple comparisons test and a Dunn's *post hoc* test: HSA-Cre^+/–^; *Terf2*^f/f^ versus *Terf2*^f/f^: *p*-value = 0.0327 and HSA-Cre^+/–^; *Terf2*^f/f^ versus HSA-Cre^+/–^: *p*-value = 0.0083. (C) Quantification of mouse fibre size repartition (all types of fibres were included). Fibres were categorized according to their surface area (<500 to >5000 µm^2^). More than 500 fibres were counted per mouse: HSA-Cre^+/–^ (young: *n* = 3, old *n* = 3), HSA-Cre^+/–^; *Terf2*^f/f^ (young: *n* = 4, old *n* = 5) and *Terf2*^f/f^ (young: *n* = 3, old *n* = 5). Student's *t*-test was performed at each size range HSA-Cre^+/–^ versus HSA-Cre^+/–^; *Terf2*^f/f^ (*) and *Terf2*^f/f^ versus HSA-Cre^+/–^; *Terf2*^f/f^ (#). (*p < 0.05, **p < 0.001, ***p < 0.001, ****p < 0.0001).

Next, we measured myofibre cross-sectional area (CSA) of *tibialis anterior* (TA) and gastrocnemius muscles. As expected, myofibre CSA of TA and gastrocnemius muscles decreased with age in HSA-Cre^+/–^ and *Terf2*^flox/flox^ mice (figure 1C; supplementary material, figure S2). Remarkably, CSA in HSA-Cre^+/–^, *Terf2*^flox/flox^ mice remained stable over time (figure 1C). These findings suggest that TRF2-compromised muscles are protected against age-related muscle atrophy, at least at the early stages of ageing (15 months).

Skeletal muscle ageing is accompanied by a strength decline. *In vivo* isometric plantar flexor *force* measurement of 15-month-old mice demonstrated that female HSA-Cre^+/–^, *Terf2*^flox/flox^ mice were marginally stronger than controls, with no significant differences observed in males (supplementary material, figure S3A,B). Fatigue protocols further revealed that HSA-Cre^+/–^, *Terf2*^flox/flox^ females outperformed other groups during early contractions (figure 2A; supplementary material, figure S3C). However, HSA-Cre^+/–^ females were more resistant to fatigue by the end of the protocol (figure 2A; supplementary material, figure S3C). Collectively, these findings support the presence of a younger-like skeletal muscle phenotype in 15-month-old female mice with muscle-specific *Terf2* deletion.

**Figure 2.**
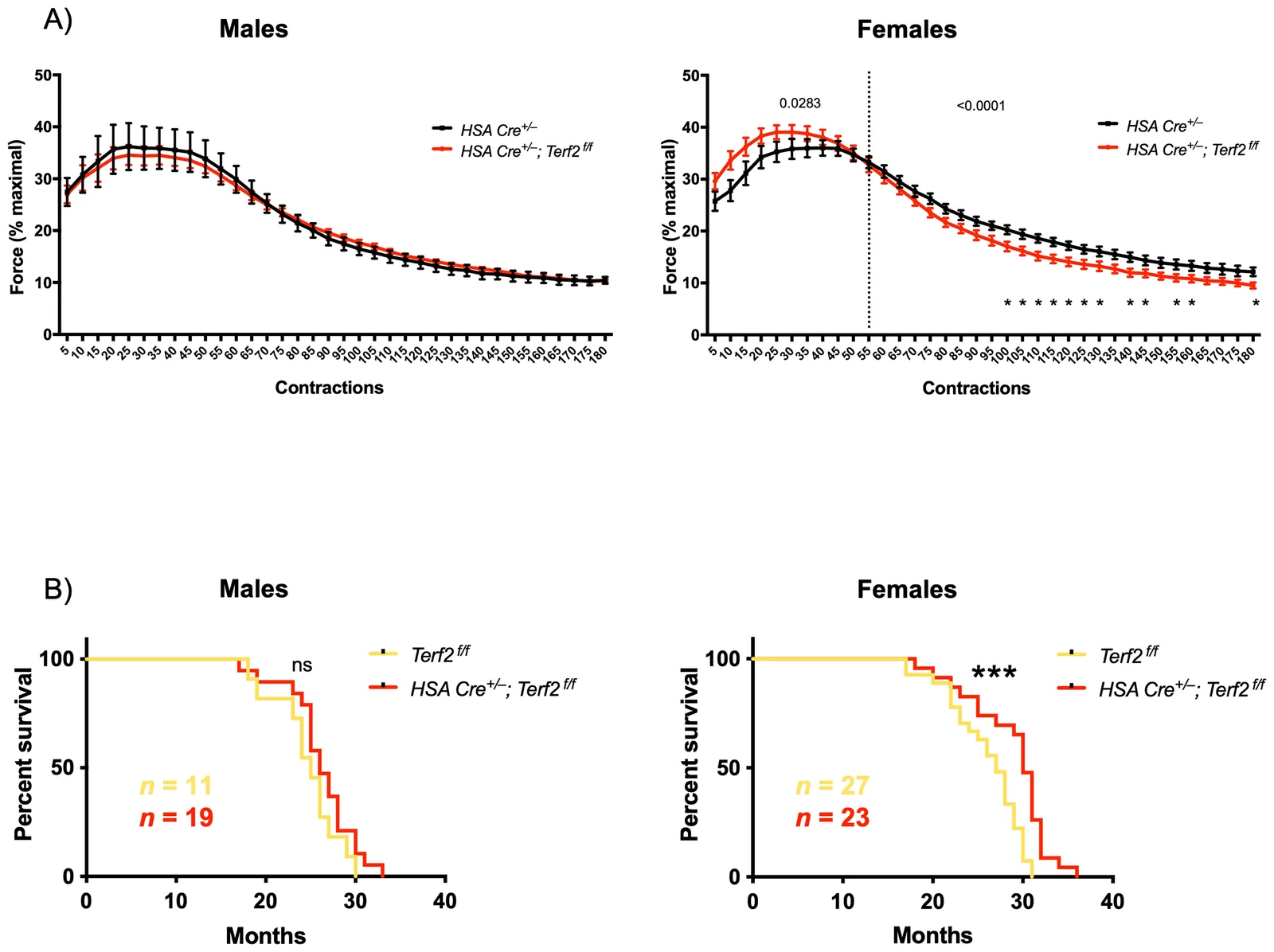
Female HSA-Cre^+/^^–^; *Terf2*^f/f^ mice are stronger and have a modest median lifespan extension. (A) Contraction-induced muscle fatigue at 30 Hz, represented as a percentage of maximal force. Left panel: males (HSA-Cre^+/–^*n* = 5 and HSA-Cre^+/–^; *Terf2*^f/f^*n* = 6 and right panel: females (HSA-Cre^+/–^*n* = 6 and HSA-Cre^+/–^; *Terf2*^f/f^*n* = 8). Each contraction point was analysed using a *t*-test (*p < 0.05). To calculate the area under the curves, data were divided into subgroups before and after the curves’ intersection. Then, the data were compared using the Mann–Whitney U test, *p*-value = 0.0283 for the first part (left) and *p*-value <0.0001 for the second part (right). (B) Kaplan–Meier survival curves of males *Terf2*^f/f^ versus HSA-Cre^+/–^; *Terf2*^f/f^, *n* = 11 and 19, respectively (left panel). Lifespan extension was observed in HSA-Cre^+/–^; *Terf2*^f/f^ female mice: 3 months median survival extension (11%) compare with *Terf2*^f/f^ female mice (right panel). *n* = 27 *Terf2*^f/f^ and 23 KO females (log-rank—Mantel–Cox, *χ*^2^ = 11.47 and *p*-value = 0.0007).

To assess the broader impact of *Terf2* inhibition on ageing, *Terf2*^flox/flox^ and HSA-Cre^+/–^; *Terf2*^flox/flox^ mice were followed through their lifespan. While the male groups showed similar median lifespans of around 25 months, female HSA-Cre^+/–^; *Terf2*^flox/flox^ mice exhibited an 11% increase in median lifespan, as compared with the *Terf2*^flox/flox^ female group (figure 2B). Female median lifespan was extended by 15% compared to males in the HSA-Cre^+/–^; *Terf2*^flox/flox^ group. By contrast, there was no female/male difference in control mice median lifespan (*Terf2*^flox/flox^) (supplementary material, figure S4). These results indicate that the loss of TRF2 in myofibres gives a slight but detectable longevity advantage specific to females, in agreement with the systemic effect of skeletal muscle on ageing [3].

Finally, we investigated the mechanisms underlying the anti-ageing effects associated with TRF2 loss. These effects are unlikely to be driven by telomere deprotection, since our previous work demonstrated that young mice lacking *Terf2* in myofibres did not exhibit deprotected telomeres [4]. Even in aged wild-type mice, we do not observe a significant increase in telomere damage-induced foci (TIFs), despite a slight rise in total DNA damage (supplementary material, figure S5A). In 15-month-old mice lacking *Terf2*, there was no significant increase in telomeric DNA damage compared with *Terf2*^flox/flox^ and HSA-Cre^+/–^ control mice (supplementary material, figure S5B). Thus, telomere deprotection does not appear to be a main driving mechanism of skeletal muscle cell ageing. Instead, the pro-oxidant environment induced by TRF2 loss may activate the telomere-protective function of FOXO3a in skeletal myofibres [5], providing a compensatory mechanism that preserves telomere integrity. This paradoxical telomere protection conferred by TRF2 loss might account for the observed resistance of these cells to senescence, with only 6% of skeletal myofibres displaying senescence signs, unlike cardiomyocytes, which show a much higher susceptibility [6,7].

To further elucidate the mechanisms underlying the protective effects of *Terf2* invalidation in skeletal muscle, we performed RNA-sequencing on the gastrocnemius*–*plantaris*–*soleus muscle group (GPS) from 8-month-old mice. Differential expression analysis revealed 78 significantly altered genes (differentially expressed genes, DEGs) in female GPS and 428 DEGs in male GPS samples (supplementary material, table S1), when HSA-Cre^+/–^, *Terf2*^flox/flox^ mice were compared with HSA-Cre^+/–^ mice. In females, DEGs were enriched for mitochondrial respiratory chain, a finding further validated by RT-qPCR analysis of *Cox5a*, *Atp5k* and *Ndufa5* (figure 3A; supplementary material, figures S6 and S7). By contrast, despite the elevated number of DEGs in males, fewer biological pathways were significantly enriched (figure 3B). The circadian rhythm pathway appeared as the most prominent, which was then confirmed by RT-qPCR analysis of circadian clock genes (*Per2*, *Npas2*, *Nrip1* and *Arntl*) (supplementary material, figure S6C). These observations are consistent with our previous work demonstrating mitochondrial dysfunction, reduced complex I activity and increased ROS in TRF2-compromised human and mouse myofibres [4], but we have not yet directly connected these transcriptional changes to functional readouts such as mitochondrial respiration and ROS production or validated the circadian gene programme in males across multiple circadian phases. Nonetheless, our current data raise the intriguing possibility that TRF2 loss engages sex-specific adaptive programmes, with mitochondrial remodelling predominating in females [4,5] and circadian rewiring in males, a hypothesis that will need to be tested directly in future work.

**Figure 3.**
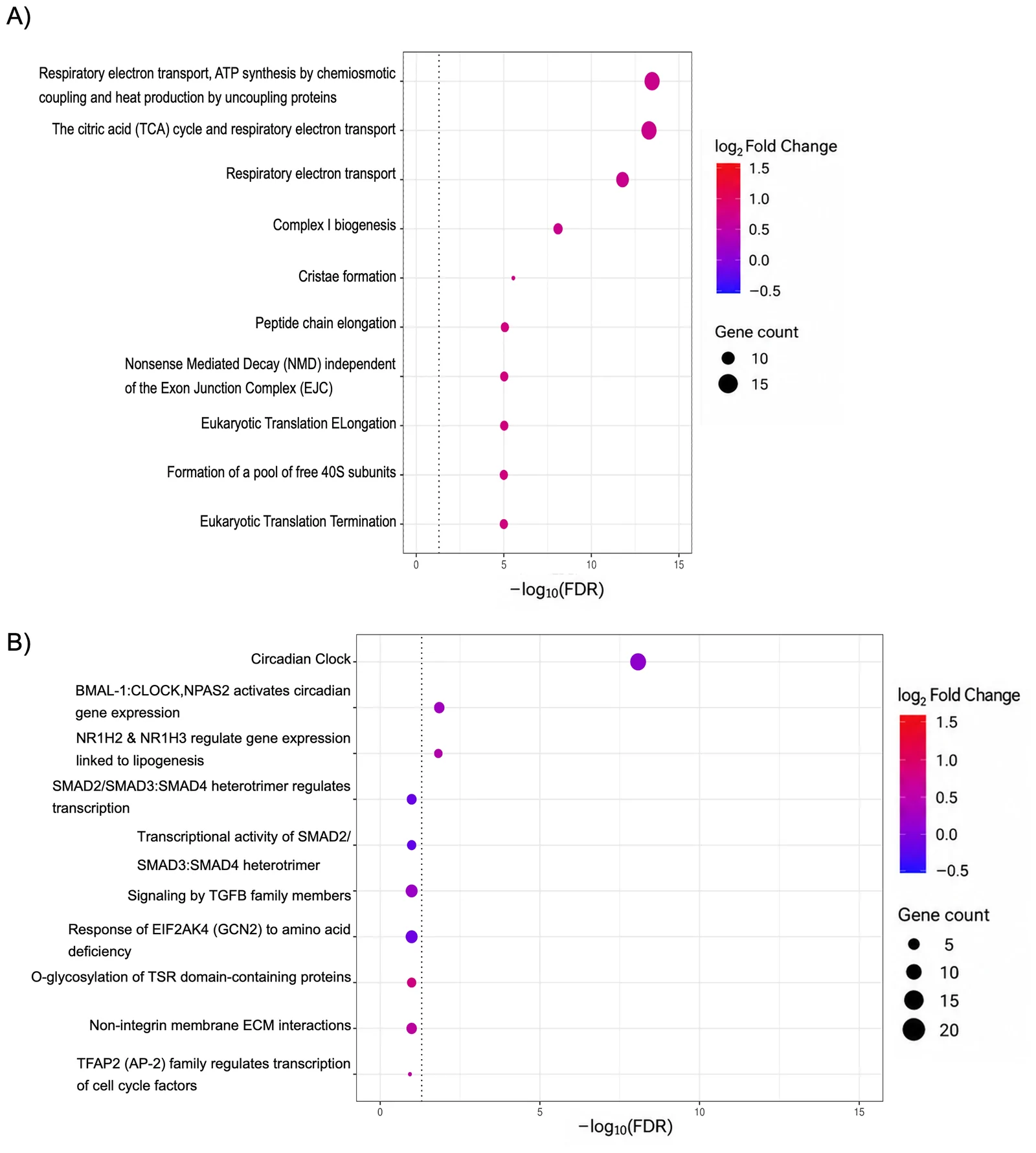
HSA-Cre^+/^^–^; *Terf2*^f/f^ mice show transcriptional differences. Reactome pathway analysis of RNA-seq differential expression data of 8-month-old females (A) and males (B) HSA-Cre^+/–^ versus HSA-Cre^+/–^; *Terf2*^f/f^ GPS. Significantly differentially expressed pathways are to the right of the dashed line. For females the top 10 significant pathways are shown. *n* = 3 HSA-Cre ^+/–^; *Terf2*^f/f^, *n* = 5 HSA-Cre^+/–^ females and *n* = 4 HSA-Cre^+/–^; *Terf2*^f/f^, *n* = 5 HSA-Cre^+/–^ males. ECM, extracellular membrane.

Our previous work showed that TRF2 loss in skeletal myofibres induces oxidative stress and recruits FOXO3a to telomeres, thereby activating a non-canonical telomere-protective and mitochondrial regulatory programme [5]. In light of evidence that FOXO3 signalling and mitochondrial function in skeletal muscle are modulated in a sex-dependent fashion [8–10] and that oestrogen–ERα pathways shape mitochondrial metabolic signalling [11], the sex-specific adaptive programme that we observe may arise because sex hormones reconfigure the TRF2–FOXO3a axis to promote a more efficient response to *Terf2* loss in female muscle. Testing this possibility will require future studies that integrate longitudinal hormonal profiling and experimental manipulation (e.g. ovariectomy and hormone replacement) with detailed analyses of TRF2/FOXO3a signalling and mitochondrial function in both sexes.

## 3. Conclusion

Our findings challenge the prevailing view that telomere dysfunction is intrinsically pro-ageing and invariably drives cellular senescence. Instead, our results demonstrate that loss of the *Terf2* gene in skeletal myofibres is associated with improved muscle maintenance during ageing, preserved muscle function and increased lifespan in females. This finding is based on HSA-Cre;* Terf2*^f/f^ mice, which lead to a loss of *Terf2* early in life, so it would be of interest to ask whether the results observed here are reproducible in an inducible, specific knockout mouse model, in which *Terf2* would be KO at different *ages.* The molecular and physiological changes observed are consistent with activation of sex-specific adaptive stress response pathways, including mitochondrial and antioxidant programmes, suggesting a potential mito-hormetic mechanism together with alternative modes of telomere protection [5,12]. While the present study does not directly establish the causal contribution of mitochondrial ROS signalling to these effects, our data support a model in which moderate telomere perturbation elicits beneficial adaptive responses that enhance cellular resilience and preserve muscle integrity. In this context, the physiological downregulation of *Terf2* previously observed in young adult skeletal muscle may represent an adaptive stress-response programme that contributes to muscle maintenance. Future studies employing targeted mechanistic interventions will be required to determine the role of mitochondrial ROS signalling and gender in this phenotype and to establish how these responses are integrated within broader muscle maintenance and ageing pathways.

## 4. Material and methods

### (a) Mice–transgenic model

Specific skeletal muscle TRF2 KO mice (HSA-Cre^+^/^–^;*Terf2*^flox/flox^) were generated as previously described [4]. First, the previously characterized TRF2 floxed mice from the Jackson laboratory (strain B6; 129P2-*Terf2*^tm1Tdl^/J) [11] were backcrossed with the C57BL/6 strain for six generations. Next, mice were crossed with the HSA-Cre^+^/^–^ strain expressing the cre recombinase under the control of the HSA gene promoter, specifically expressed in striated muscle fibres.

One-, three-, 12- and 15-month-old C57BL6/J mice were directly ordered from Charles River and maintained for one week of adaptation within the IRCAN Experimental Core Facility (Nice) before organ collection.

### (b) Fibre size and nucleus position

Mice were sacrificed and fresh muscle was dissected: GPS and TA and then flash-frozen in cold isopentane steam (dry ice) in an optimal cutting temperature compound block. Transverse frozen sections (10 µm) of each muscle from mice were prepared using a Leica cryostat and mounted onto SuperFrost Slides. Full tissues and slides were kept at –80°C for storage.

For staining, muscle slides were melted at room temperature for 30 minutes and fixed for 10 minutes with 4% paraformaldehyde. Next, slides were washed three times in PBS 1X for 3 minutes and permeabilized in 0.5% Triton X-100 in PBS for 1 hour and washed again in PBS 1X. After 1 hour of blocking using a solution of M.O.M IgG diluent (Vector Laboratories, Burlingame, USA) at room temperature, slides were washed twice in PBS 1X for 5 minutes and incubated with M.O.M protein diluent solution for 5 minutes. Next, slides were incubated with primary antibodies in M.O.M protein diluent (1 : 20) overnight at 4°C. Each slide was treated simultaneously with four conditions separated using a Dako pen (Agilent); MyHC-I (Isoform BA-D5, DSHB, University of Iowa), MyHCIIa (Isoform SC-71, DSHB, University of Iowa) specific to type I and type IIa fibres, respectively, as well as combined MyHC-I + MyHC-IIa and no primary antibody. After three washes of 5 minutes each in PBS 1X, slides were incubated for 40 minutes in M.O.M protein diluent containing the secondary antibody (Alexa 488 Donkey anti-mouse; 1 : 200) and washed three times for 5 minutes in PBS 1X. Lastly, slides were mounted with Vectashield containing DAPI (Vector Laboratories, Burlingame, USA). Images were taken using an Axio Imager 2 and a 20X lens (Zeiss).

Muscle fibre size was determined using Lmaris software. More than 1000 fibres were analysed per mouse, and three to five mice per genotype and age.

For peripheral or central nucleus position, 100 fibres were counted per mouse (three to six mice per genotype and age).

### (c) *In vivo* isometric force measurement

Mice were initially anaesthetized in an induction chamber using 4% isoflurane. The right hindlimb was shaved before an electrode cream was applied at the knee and heel regions to optimize electrical stimulation. Each anaesthetized mouse was placed supine in a strictly non-invasive ergometer (NIMPHEA_Research, AII Biomedical SAS, Grenoble, France) offering the possibility to electrically stimulate the plantar flexor muscles of the mouse and to record the resulting force production. Throughout a typical experiment, anaesthesia was maintained by air inhalation through a facemask continuously supplied with 2.0–2.5% isoflurane. The cradle also includes an electrical heating blanket in order to maintain the animal at a physiological temperature during anaesthesia. Electrical stimuli were delivered through two electrodes located below the knee and the Achilles tendon. The right foot was positioned and firmly immobilized through a rigid slipper on a pedal of an ergometer, allowing for the measurement of the force produced by the plantar flexor muscles. The right knee was also firmly maintained using a rigid fixation in order to optimize isometric force recordings. Monophasic rectangular pulses of 0.2 ms were delivered using a constant-current stimulator (Digitimer DS7AH, Hertfordshire, UK; maximal voltage: 400 V). The force–frequency curves were determined by stepwise increasing stimulation frequency, with >30 s between stimuli in order to avoid effects owing to fatigue. After a 3-minute recovery period, force was assessed during a fatigue protocol consisting of 180 stimulation trains delivered at 30 Hz (300 ms on, 700 ms off). The corresponding force was averaged every five contractions and was expressed in relation to maximal force. The force signal was sampled at 1000 Hz using a PowerLab system and LabChart software (ADInstruments).

### (d) RNA sequencing

Mouse skeletal muscles were snap-frozen in liquid nitrogen and preserved at –80°C. Total RNA was extracted using Tris Reagent (T9424—Merck), according to the manufacturer's instructions. In brief, muscles were incubated in Tris Reagent in ceramic bead tubes (KT03961-1-003.2 Precellys) and dissociated with a Precellys tissue homogenizer. After the addition of 200 µl chloroform, the muscles were centrifuged for 15 minutes at 12000 g (4°C). The aqueous phase was transferred; RNA pellet was precipitated with 500 µl isopropanol and centrifuged again. Next, the RNA pellet was washed twice in 75% ethanol. RNA quantification was determined by a NanoDrop 1000 spectrophotometer (Thermo Scientific), and RNA quality was assessed with an Agilent 2100 Bioanalyzer. Paired-end sequencing (read length: 2 × 150 bp) was performed by NovoGene using an Illumina NovaSeq 6000 sequencer.

### (e) qPCR

After total RNA extraction, reverse transcription (RT) was performed with 500 ng RNA using the high-capacity RNA-to-cDNA kit (Thermo Scientific). Each qPCR contained 5× diluted cDNA, 0.2 µm primers and SYBR Green Master Mix (Roche, 4913914 001). Quantitative RT-PCR (qRT-PCR) was performed in triplicate using FastStart universal SYBR Green Master Mix (Roche). Melting curves were analysed (SYBR Green) to exclude non-specific amplification products. We confirmed amplicon size at least once on agarose gels. Crossing-threshold values were normalized to the *36B4* housekeeping gene.

### (f) Telomere damage-induced foci

GPS slides were prepared as mentioned above. After fixation, slides were permeabilized with 0.5% Triton X-100 for 20 minutes and dehydrated in increasing concentrations of ethanol for 3 minutes (50, 70 and 100%). Hybridization of the peptide nucleic acid (PNA) telomeric probe was performed for at least 2 h at RT after denaturation (5 minutes) in 70% formamide, 10 mM Tris pH 7.2 and 1% blocking solution (Roche) at 80°C. Then, slides were washed in a 70% formamide, 10 mM Tris pH 7.2 solution for 30 minutes, followed by washes with 150 mM NaCl and 50 mM Tris pH 7.5 for 15 minutes. Next, slides were incubated for 1 h with blocking buffer (3% BSA and 0.3% Triton X-100) and incubated overnight at 4°C with 53BP1 antibody, rabbit polyclonal (NB 100-305, 1 : 200, Novus Biologicals). Slides were then washed three times with 1X PBS and incubated for 1 h with the corresponding secondary antibody. Finally, slides were prepared with a DAPI-containing mounting solution (Vectashield, Vector Laboratories).

Pictures were taken using a DeltaVision Elite system (GE). An average of 70 stacks and 30 to 40 nuclei were taken per condition. Images were then analysed using FIJI (ImageJ). The total number of 53BP1 foci was determined, and telomere damage-induced foci (TIFs) were determined manually by doing a stack-by-stack screening looking for at least one colocalizing pixel (yellow). Animal age and genotype were blinded to the experimenter during image analysis to prevent bias in data acquisition and quantification.

## 5. Statistical analysis

GraphPad Prism 7 software was used to generate graphs and to perform statistical analysis. *p*-Values were obtained using either the Mann–Whitney U test or the Kruskal–Wallis test. Differences were considered statistically significant when *p *< 0.05, as follows: *p < 0.01, **p < 0.001, ***p < 0.0001. Absence of statistical annotation indicates non-significance.

## Supplementary Material



## Data Availability

The raw sequencing data (FASTQ files) and processed gene count matrices generated in this study have been deposited in the NCBI Gene Expression Omnibus (GEO) under accession number: GSE325832 (https://www.ncbi.nlm.nih.gov/geo/query/acc.cgi?acc=GSE325832). Supplementary material is available online [13].

## Theme

One contribution of 21 to a theme issue ‘Developmental circuits in ageing’.
