# Supplementary material for: Female-specific anti-ageing effects of TRF2 inhibition in skeletal myofibres
Source: Philos Trans R Soc Lond B Biol Sci. 2026 Nov 12;381(1963):20250336. doi: 10.1098/rstb.2025.0336 (PMC13755912; doi:10.1098/rstb.2025.0336)

Supplemental figure 1

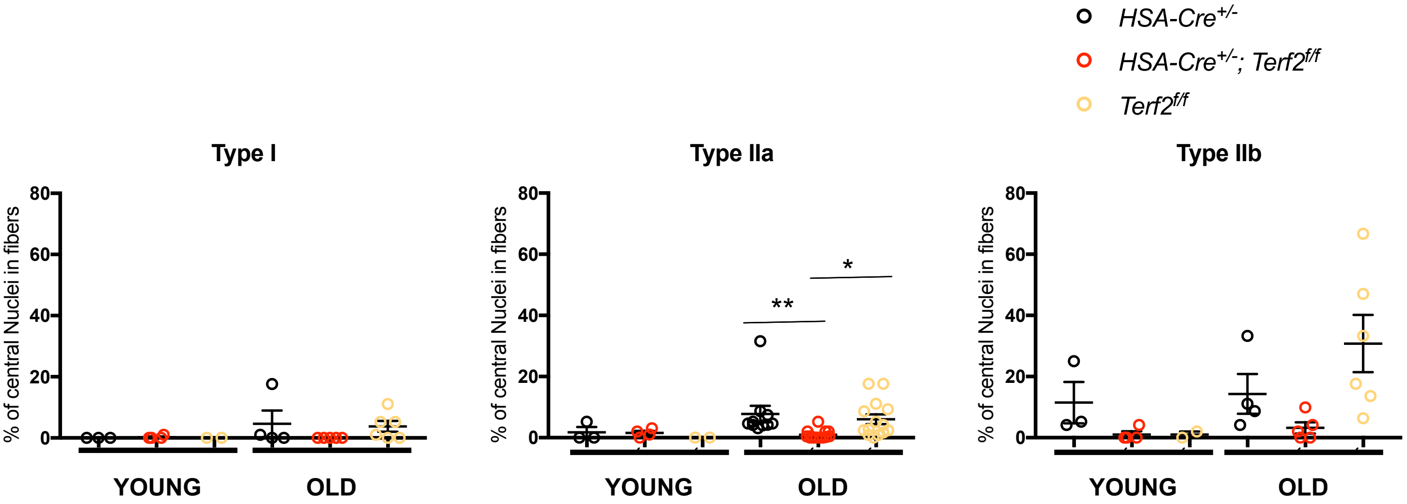

Supplemental figure 2

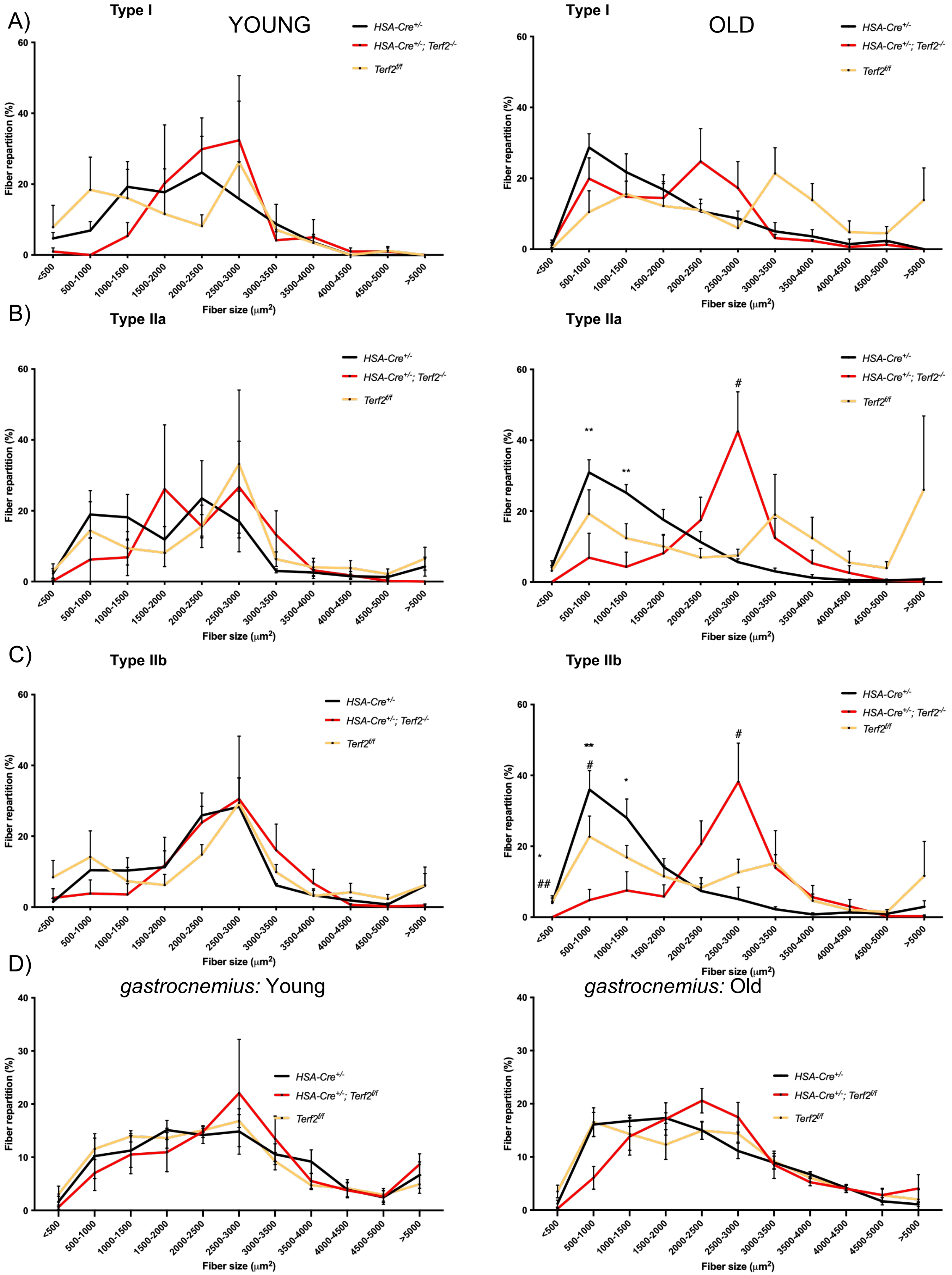

Supplemental figure 3

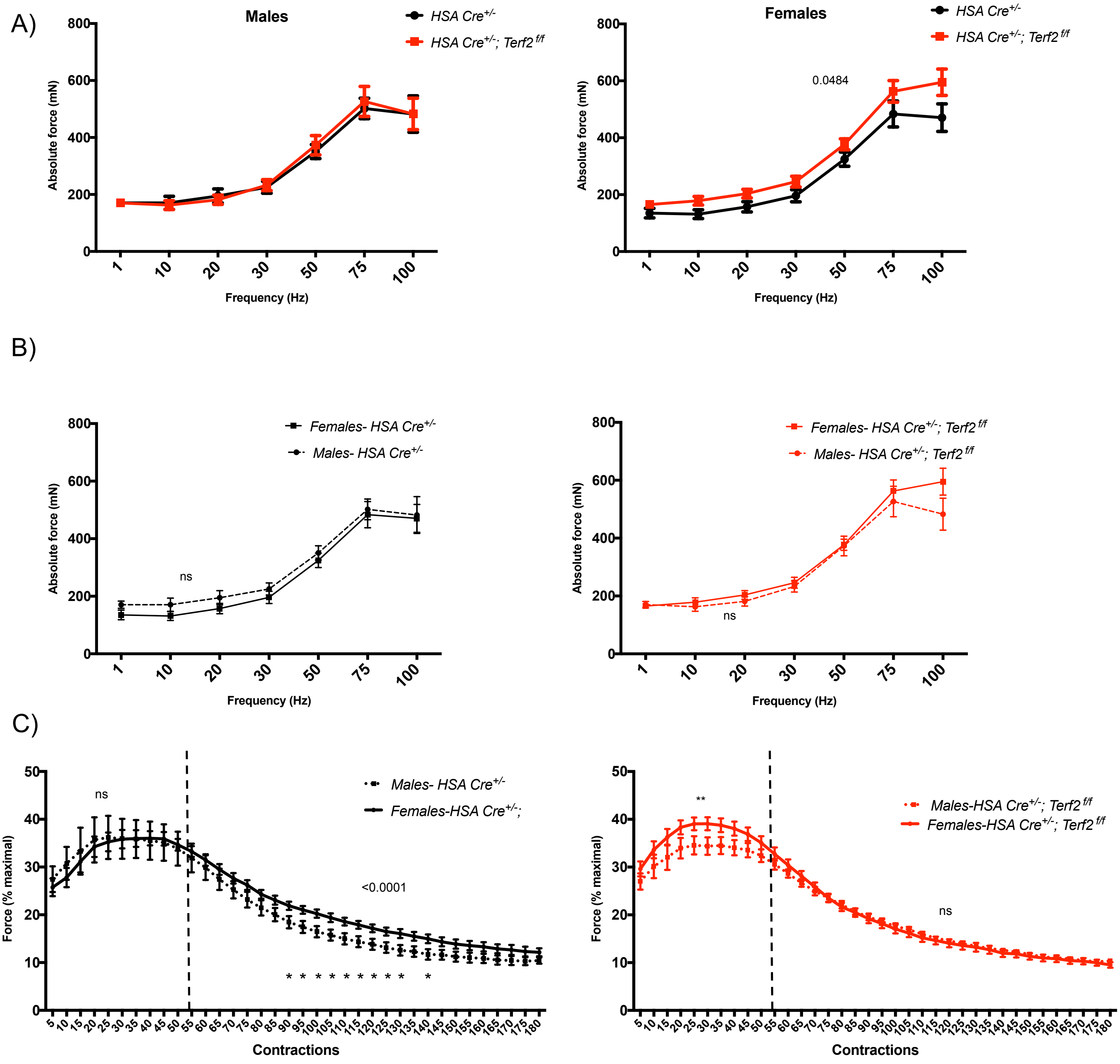

Supplemental figure 4

A)

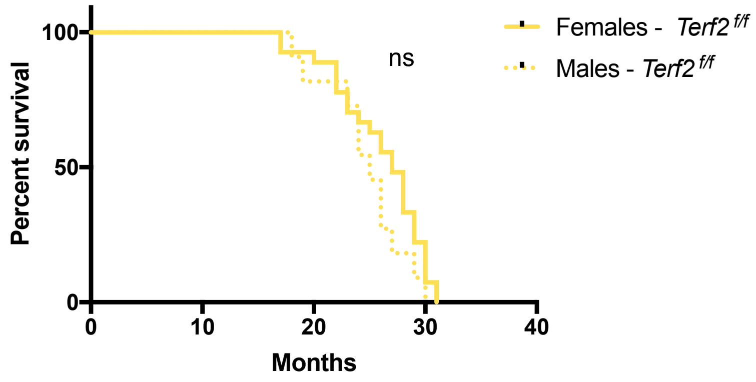

B)

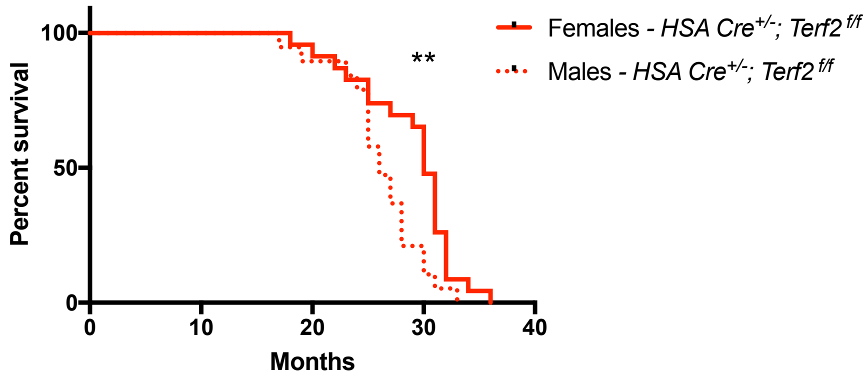

C)

Sex / Genotype	Females Terf2 flox/flox	Males Terf2 flox/flox	Females HSA-Cre+ / - ;Terf2flox/loxf	Males HSA-Cre+ / - ;Terf2flox/loxf
Median survival (months)	27	25	30	26

Supplemental figure 5

A)

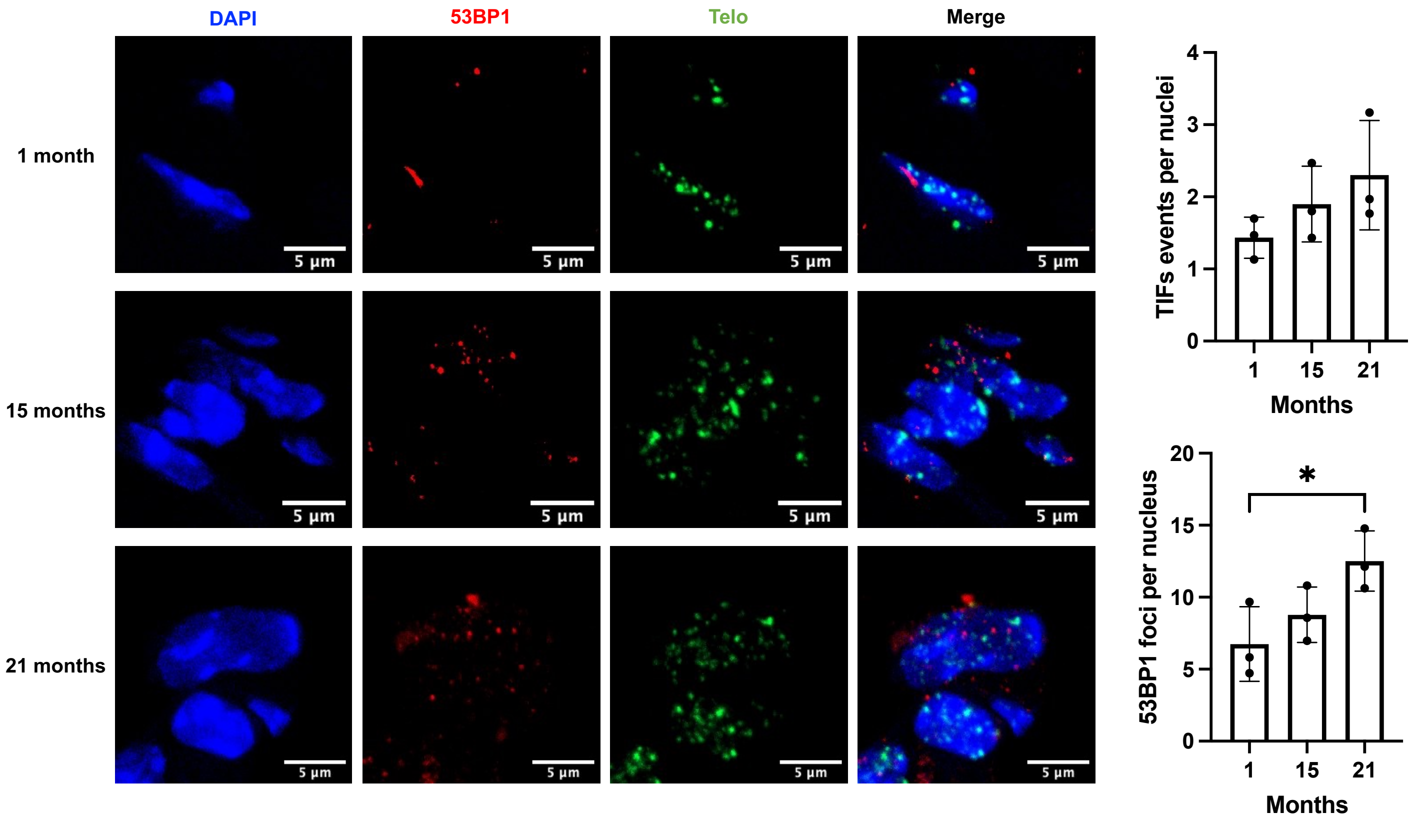

B)

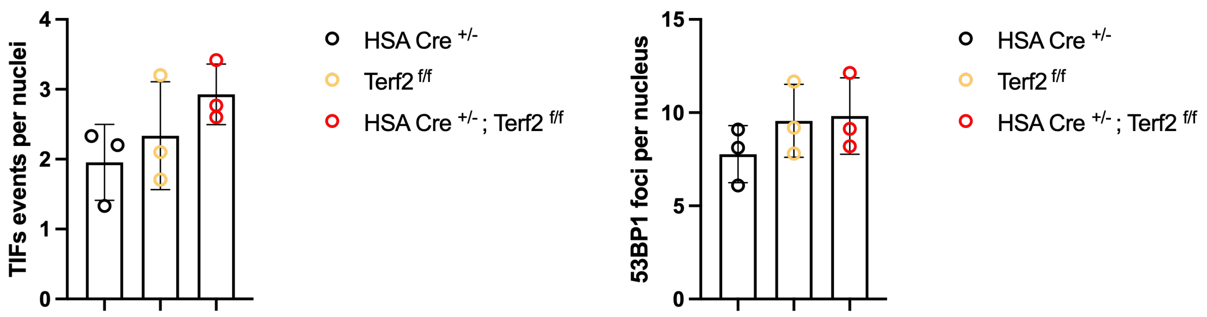

Supplemental figure 6

A)

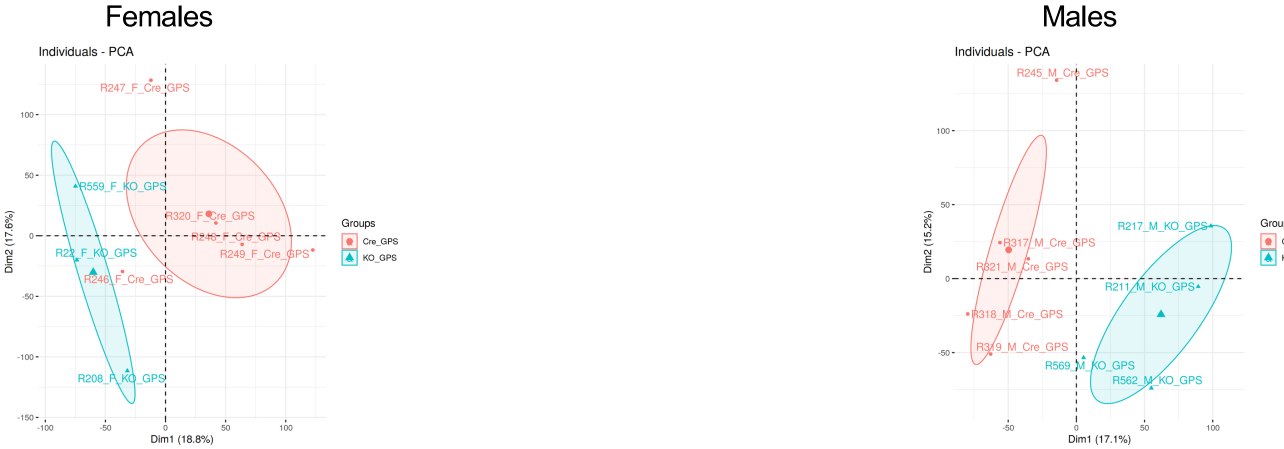

B)

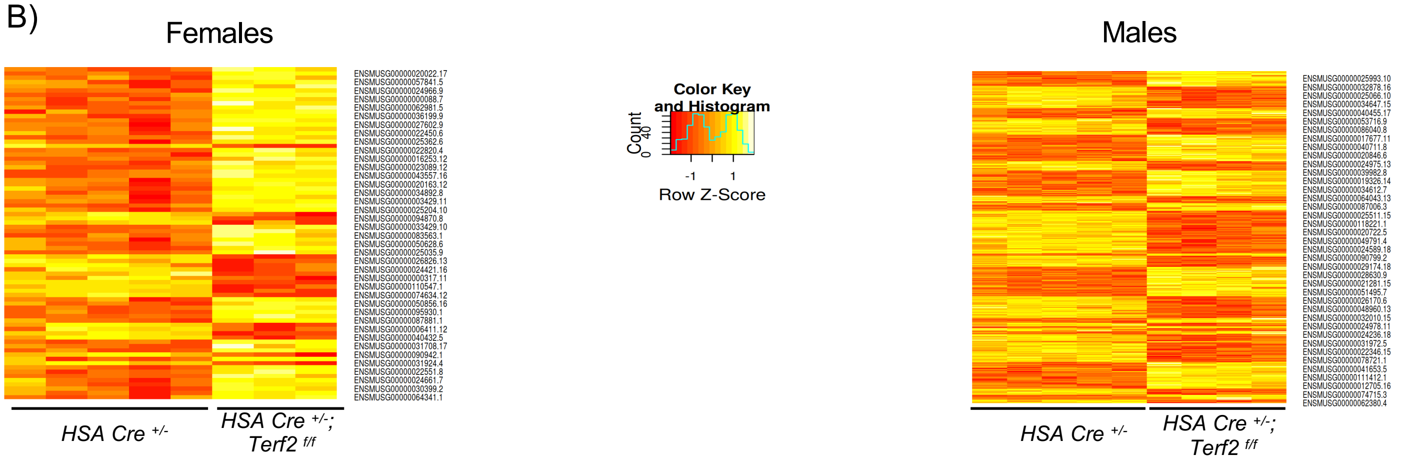

C)

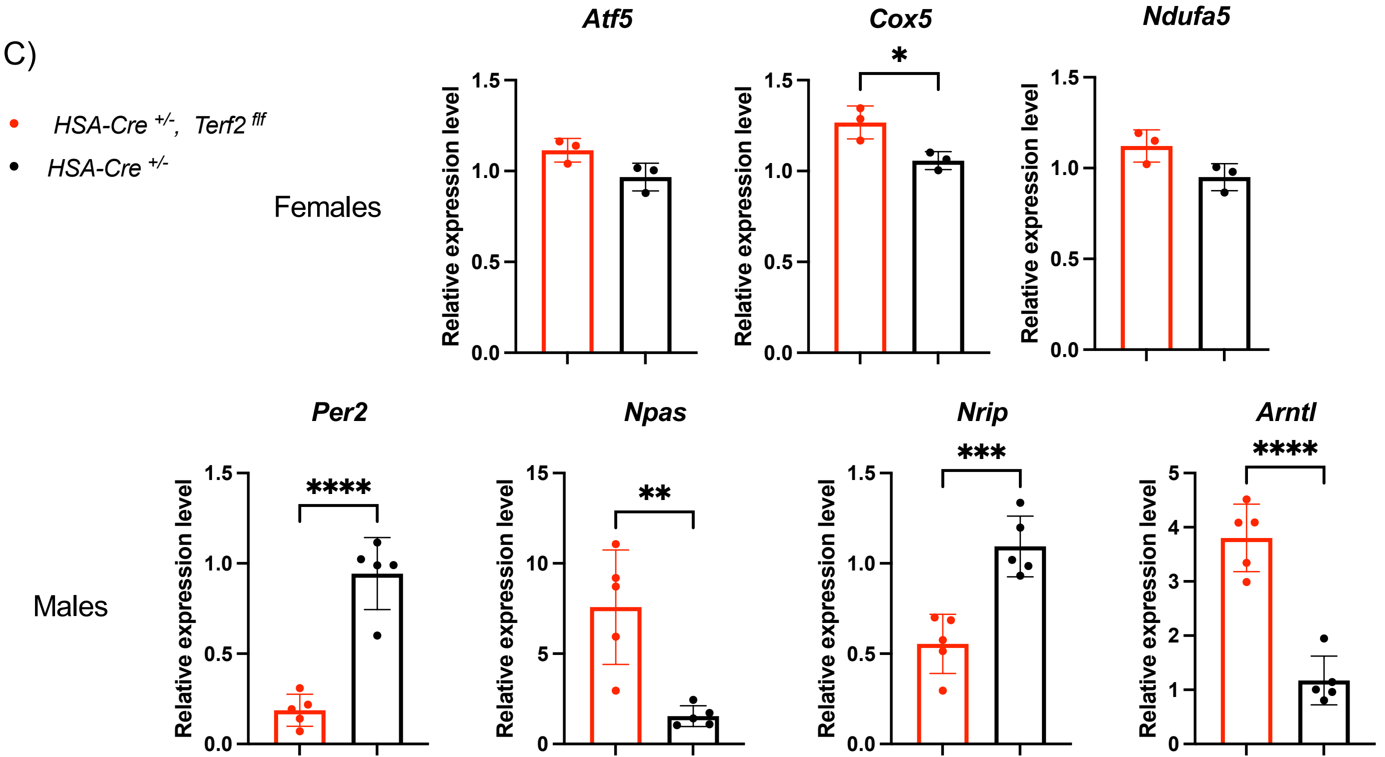

Supplemental figure 7

A)

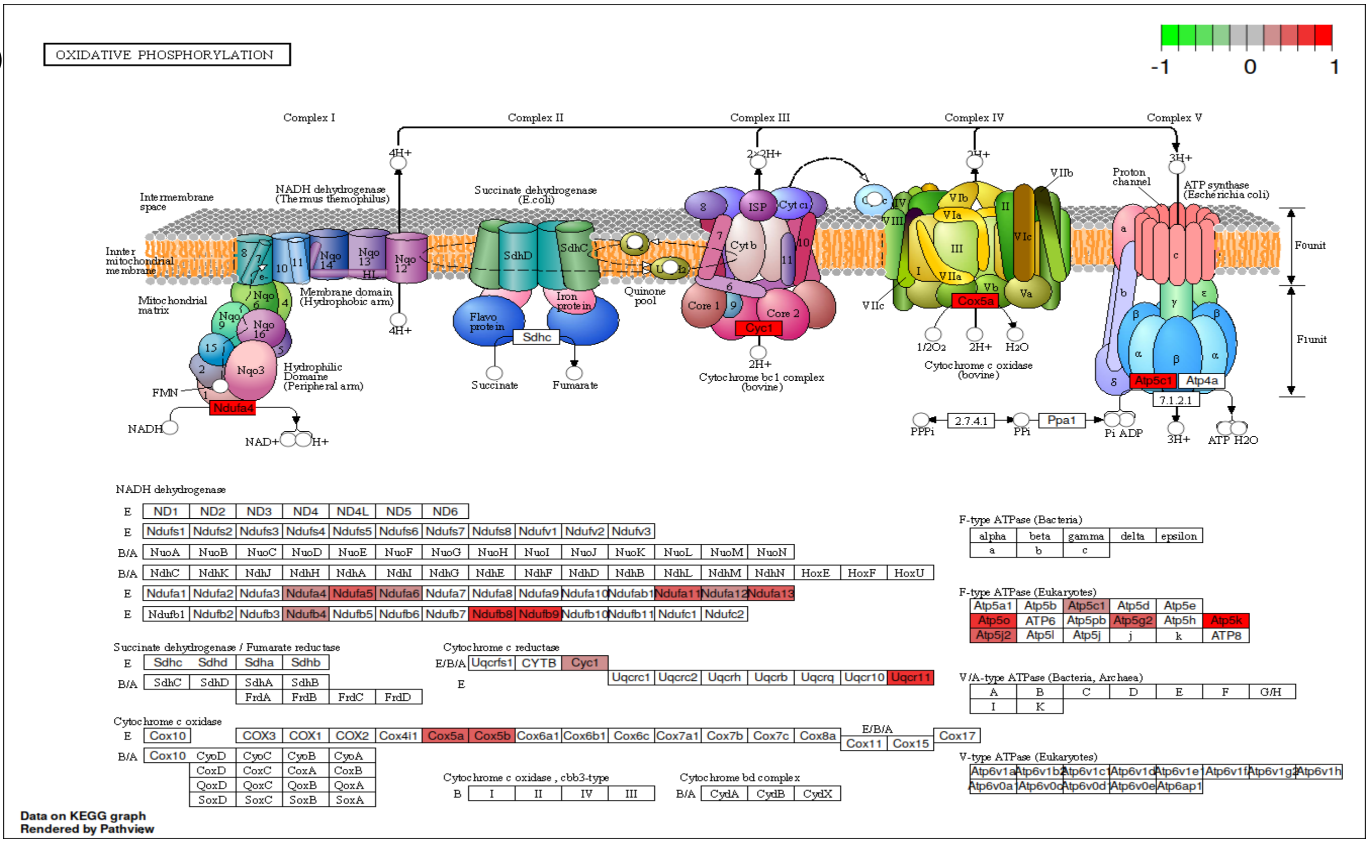

B)

KEGG

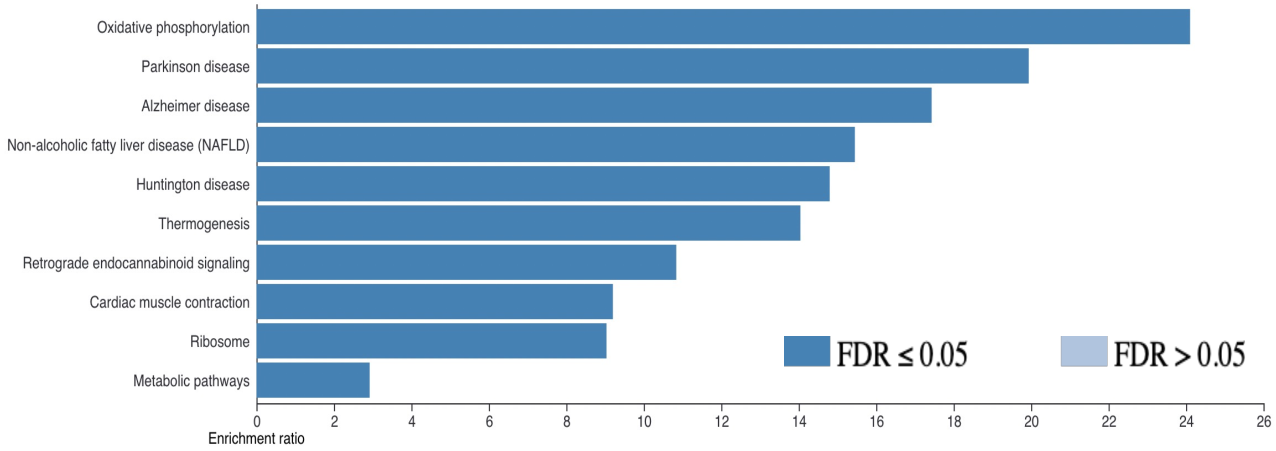

Wikipathway

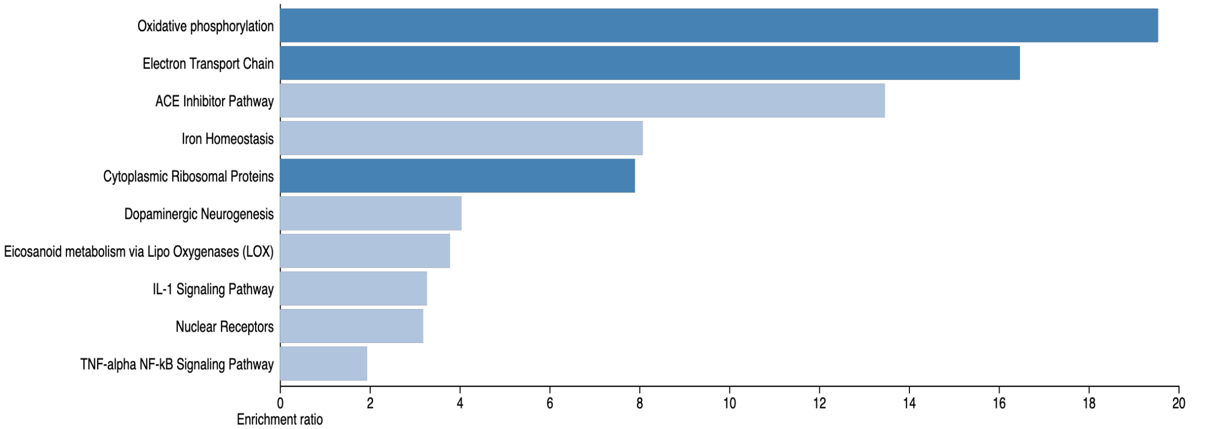

Supplement: Supplementary file 1 [file rstb.2025.0336.zip › rstb.2025.0336/rstb20250336_si_001.pdf]
